# AI enhances drug discovery and development

**DOI:** 10.1093/nsr/nwad303

**Published:** 2023-11-28

**Authors:** Fang Bai, Shiliang Li, Honglin Li

**Affiliations:** Shanghai Institute for Advanced Immunochemical Studies and School of Life Science and Technology, ShanghaiTech University, China; Lingang Laboratory, China; Innovation Center for AI and Drug Discovery, East China Normal University, China; Lingang Laboratory, China; Innovation Center for AI and Drug Discovery, East China Normal University, China; Lingang Laboratory, China

The advent of the third wave of artificial intelligence (AI) heralds a new era in drug discovery. AI-powered techniques can be characteristic of identifying features that are difficult for humans to interpret from big and high-dimensional data in biomedical research. That's why there is so much enthusiasm for AI in drug discovery, not only in academia but also in industry. Booming AI tools are revolutionizing nearly every stage of the drug discovery process, indicating the substantial potential to transform the speed and economics of the industry. Our previous review provided a comprehensive overview of developments in this area [1]. Due to space limitations, we focus here only on the frontiers of the five technologies or phases of drug development mentioned below (Fig. 1).

**Figure 1.**
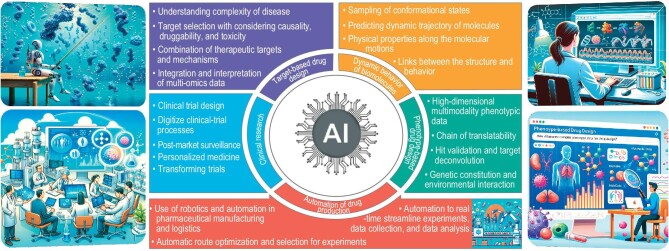
Example modules and their challenges in AI enhanced drug development pipeline. Cartoon figures were drawn with the assistance of DALL·E 3 [10].

## DEEPENING AI IN TARGET-BASED DRUG DEVELOPMENT

Target-based drug development (TDD) has been the predominant approach to new drug discovery for more than 30 years, but many candidates still fail in clinical trials due to efficacy or safety concerns, leading researchers to look at the complexity of disease from more than just a target perspective. Multi-omics data, including genomics, proteomics, metabolomics, and epigenetics, from both the static and dynamic sides, are helping to capture the complexity of disease. AI has shown promise in extracting useful knowledge from vast high-dimensional, and even noisy databases, such as integrating complex multi-omics data by building networks that simulate disease-causing pathways [2], or mapping knowledge about target-diseases from the literature to construct linkages between target diseases and bioactive compounds [3]. Despite the advantages that data integration brings, the lack of interpretability and consistency of validation data across databases are the main challenges. Presenting on-target modulating efficacy and potential side effects on one page will be a trend in the near future to consider a new target.

Structure-based target identification is another type of TDD that has been employed for decades. However, due to very limited coverage of the target space and methods to evaluate the ‘druggability’ of proteins, the accuracy still cannot meet the requirements. Therefore, this is another issue that can be improved by AI.

## CAPTURING DYNAMIC BEHAVIOR OF MOLECULES WITH AI

Determining the different conformational states as well as the continuous transition pathways between them for a macromolecule is a prerequisite for understanding the biological function of the molecule and the basis for drug design. Such tasks can be performed by molecular simulation methodologies. However, the fundamental difficulties, such as limited conformational search capability and high computational cost, greatly hinder their application to large and complex biological macromolecules. Recently, the introduction of AI algorithms has revolutionized the field of protein structure prediction, and several methods have been developed, such as AlphaFold, RoseTTAFold, and ColabFold [4]. In addition to protein structure, the newly announced AlphaFold3 model also predicts the structure of ligands, nucleic acids and post-translational modifications. Advances in biomolecule prediction methods are also significantly boosting progress in the development of biologics. Nevertheless, methods for predicting dynamic behavior or conformational ensemble are still in their infancy. In the context of this topic, several problems on the physical model-based conformational retrieval side could be addressed by AI, as shown by Noé *et al.* [5], such as quantum mechanical energy and force prediction, the extraction of free energy surfaces and kinetics, molecular equilibrium structure generation, and thermodynamic calculations. On the other hand, conformation-to-conformation generators using end-to-end AI models will play an important role. Some methods using generative AI, e.g. GAN, diffusion model, etc., to predict protein structures or even dynamic conformations have recently been reported [6,7].

## TAKING AI TO PHENOTYPIC DRUG DISCOVERY

Phenotypic drug discovery (PDD) is a technology for discovering biologically active agents that can directly alter the phenotype of a cell or organism. Since the available chemical and biological space is limited to known current compound libraries and disease model systems, phenotypic screening provides the opportunity to identify molecules that act on multiple targets, which is referred to as polypharmacology. The superiority of PDD lies in that it addresses the complexity of diseases that are poorly understood or where the disease-related gene is ‘undruggable’. To date, there have been rapid advances in several technologies for PDD, e.g. induced pluripotent stem (iPS) cells, gene editing tools, organoids and imaging assays, and animal models of diseases, e.g. zebrafish, mouse, etc. [8] AI has made inroads in drug development, but applications in phenotypic traits are still rare. AI-assisted phenotypic screening can analyze images to capture both large morphological changes in cells and small morphological changes that are difficult to quantify visually. On the other hand, other issues related to PDD itself needed to be addressed: (1) limited phenotypic data, including data size, quality, and multimodality, etc.; (2) the ‘chain of translatability’ linking the primary phenotypic assay at the beginning to efficacy in the patient at the end is always unclear [9]; (3) powerful AI algorithms or data mining methods to deal with the high-dimensional multimodality phenotypic data and tame the data deluge.

## REALIZING AUTOMATION OF DRUG GENERATION WITH AI

Exploding and digitalized data, rapidly evolving AI, and new types of technologies are bringing transformative benefits to medicine and pharmaceutical research. Now they’re jumping into the robotics race. With robots in the labs, experiments would be designed and performed faster. High-quality data are produced and fed back to such AI-controlled robots to strengthen their capabilities to make more accurate hypotheses or to validate existing hypotheses. Although a few of robots have already been developed for different stages of drug development, e.g. the target fisher Eva, the lead compound hunter Alex, and RoboRXN, a compound synthesis robot, an automated AI-powered platform for drug development is still a long way off due to the complexity of the task.

## BOOSTING DRUG DEVELOPMENT FROM THE CLINICAL POINT OF VIEW

The future of clinical development is about to undergo a major transformation as the convergence of large new digital data sources and the computational power to identify clinically significant patterns in data using efficient AI algorithms, lead to a redesign of clinical trials to accelerate the drug development process, including but not limited to the following areas: identifying appropriate cohorts by mining medical records and social medica content, speeding up recruitment stages by simplifying complex entry criteria to make them more accessible to potential candidates, developing novel patient-centered endpoints, and collecting and analyzing real-world data.

In recent years, the use of AI in drug research has made great progress. At the same time, however, there are always new challenges in this field, e.g. the mismatch between the complex biomedical problem and the available data and knowledge, the limitations of technologies and hardware, etc. Nevertheless, AI-assisted drug development holds enormous potential to improve access to new medicines.
